# Supplementary material for: Implementing a multi-cycle datapath with Liquid Marbles
Source: PLoS One. 2024 Aug 20;19(8):e0309066. doi: 10.1371/journal.pone.0309066 (PMC11335127; doi:10.1371/journal.pone.0309066)
Supplement: Attachment — Submitted filename: ResponseToReviewers.pdf [file pone.0309066.s001.pdf]

Answers to reviewers' comments for the paper

Implementing a Multi-cycle Datapath with Liquid Marbles

We thank the reviewers for highlighting the issues in the paper and providing valuable suggestions. We have made an effort to better explain our choices and the ideas behind the proposed circuits. We carefully considered all the requests and suggestions and have addressed them as satisfactorily as possible.

Reviewer #1:

Major comments:
1. Setups of the LM logic circuits are not illustrated in detail. For example, where is the input or output in Figure 1?

ANS: We have added input definitions for Figure 1, the only one without labels. For Figure 1(a), the newly added labels have been mentioned in the text, lines 66-68.

2. Is it necessary to use LM in the proposed logic circuits? If the answer is no, the proposed logic circuits must be new for the BBM system to keep the novelty of this work.

ANS: The main reason behind the use of LMs over BBM mainly concerns the possibility of merging, and, consequently, the simplified circuits that can be obtained in this case. The advantages of this have been highlighted in lines 69-76.

Reviewer #2: The presents a nice idea, however, it definitely needs a revision.

Remarks.
1. Liquid marbles are not stable. The are connected to atmosphere and they evaporate. This will impact the suggested computation via liquid marbles.

ANS: We considered the evaporation issue, cited related references and explained how the problem can be faced in our solution. See highlighted text on rows 542-556.

2. What is the estimated single computation time?

ANS: In order to better analyse the problem just mentioned, we have implemented the study concerning computation times with a more complete Table, placed after row 556 and cited

on rows 537-541. An analysis was also made on the minimum and maximum speed, in lines 76-89. Considerations related to LMs speed have been made through the whole paper.

3. Elastic properties of liquid marbles should be considered, see:
Pogreb, R., Balter, R. et al. Elastic properties of liquid marbles. Colloid Polym Sci 293, 2157–2164 (2015).

ANS: We considered the suggested reference, and modified the paper accordingly. We further analyzed the elastic properties of LMs, their deviations from BBM, and the impact on the interaction gate. The related discussion have been mainly added in rows 161-170.
